# Chronic Infective Endocarditis Linked to Staphylococcus epidermidis Infection of a Pacemaker Lead: A Case Report

**DOI:** 10.7759/cureus.99028

**Published:** 2025-12-12

**Authors:** Osama S Abdalla, Ghada Idris, Darshani Ekanayake, Laila Khallaf, Charlotte Adjepon

**Affiliations:** 1 Acute Medicine, Shrewsbury and Telford NHS Trust, Telford, GBR; 2 General Medicine, Shrewsbury and Telford NHS Trust, Telford, GBR

**Keywords:** antibiotics, endocarditis, pacemaker, staphylococcus epidermidis, transoesophageal echocardiography

## Abstract

The diagnosis and management of pacemaker-related infective endocarditis present significant challenges, with limited available data. Accurately attributing a systemic infection to pacemaker endocarditis can be difficult, particularly in identifying vegetations and obtaining positive blood cultures from patients who have undergone non-specific antibiotic therapy. Moreover, such infections may manifest long after pacemaker implantation. Herein, we present a male patient in his 70s, with a history of pacemaker placement, who was admitted with a three-month history of fever and chills, having already completed two courses of empirical antibiotics prior to admission. Upon hospital admission, he was treated for an infection of unknown origin with intravenous antibiotics. Initial laboratory evaluations indicated leucocytosis and elevated C-reactive protein levels; however, blood cultures and infectious serologies returned normal results. A CT scan of the abdomen and pelvis was deemed unremarkable, and transthoracic echocardiography (TTE) also yielded normal findings. The empirical antibiotic regimen was discontinued, leading to three sets of blood cultures being subsequently positive for coagulase-negative *Staphylococcus epidermidis*. A transoesophageal echocardiography (TOE) was performed, revealing vegetation on the pacemaker lead. The patient received a triple antibiotic therapy and underwent device removal; subsequent blood cultures were negative following a four-week antibiotic course. A new pacemaker was implanted, and the patient has since remained asymptomatic. This case illustrates that coagulase-negative *Staphylococcus epidermidis* can infect pacemaker leads even long after installation, potentially leading to an indolent course of infective endocarditis that is difficult to diagnose and manage. Consequently, clinicians should maintain a high index of suspicion for pacemaker infective endocarditis in patients presenting with prolonged fever.

## Introduction

Pacemakers and other implantable cardiac electronic devices (ICEDs), which include implantable cardioverter‐defibrillators (ICDs) and cardiac resynchronisation therapy systems, are becoming increasingly common in cardiology practice, in part because of expanding indications, ageing populations, and more frequent device revisions and replacements [1]. However, with their use comes a significant and potentially life‐threatening complication: infection of the extra‑ or intra‑cardiac components of the device (generator “pocket”, leads, or those parts that contact cardiac tissue) [2]. Diagnosing these infections can be especially challenging because they often present with vague or indolent signs; standard investigations (blood cultures, device‑site inspection, transthoracic echocardiography (TTE) may lack sensitivity, especially for lead or endocardial involvement [1].

Management of ICED infections is equally difficult. While antimicrobial therapy is necessary, it is almost never sufficient by itself when there is involvement of the leads or intravascular hardware. The current guidelines from international societies, including European, American, and UK bodies, strongly recommend complete removal of the device (generator and all leads) as soon as it is feasible [2]. Delays in removal, incomplete extraction, or conservative management without device explantation are associated with much higher rates of relapse, systemic infection, and death [3].

However, device removal (particularly lead extraction) is not without risk. It can be a major invasive procedure, often performed in specialised centres. Complications range from minor to catastrophic (vascular injury, cardiac tamponade, superior vena cava laceration, damage to cardiac valves), and mortality related to the procedure, although in experienced centres, is relatively low [3]. Furthermore, even with successful removal and appropriate antibiotic therapy, patients retain a substantial mortality risk, both in-hospital and over longer-term follow‑up [4].

## Case presentation

The patient was a male in his 70s who had been encountering cold and shivering episodes two to three times a week for the past three months. These episodes have a duration of 30 to 90 minutes, with subsequent occurrences of excessive heat and a temperature rise to 38 degrees Celsius. Initially, the patient received two courses of oral antibiotics from his general practitioner, followed by intravenous antibiotics (piperacillin with tazobactam) upon presentation at the hospital. There were no reported incidents of loss of consciousness, tongue biting, eye-rolling, or incontinence. The patient resided with his spouse, owns a pet dog, and has no interaction with farm or domesticated animals. His medical history included a pacemaker for a 2:1 heart block, which was replaced a year ago, in addition to a diagnosis of glaucoma, hypertension, and recurrent epididymitis. His main concerns were the rigors, and he wanted to know the underlying cause of his symptoms.

Upon admission, the patient was found to be haemodynamically stable, and the physical examination did not reveal any noteworthy findings. Notably, no signs of infected endocarditis or indications of pacemaker pocket infection, such as erythema or discharge, were observed.

Clinical findings

On examination, he had a fluctuating temperature; other vital signs were quite stable. Heart sounds were normal to auscultation, with no murmurs or added sounds. The chest was clear, and there was no clinical evidence of consolidation. The abdomen was soft and lax, with no palpable organs. The leg showed no evidence of cellulitis. Urine dip was negative for nitrite.

Diagnostic assessment

The complete blood count (CBC) indicated leucocytosis with neutrophilia, along with elevated C-reactive protein levels that fluctuated. Three sets of blood cultures initially yielded negative results, but subsequent repeated blood cultures tested positive for Staphylococcus epidermidis. Urinalysis revealed normal microscopy, and respiratory polymerase chain reaction (PCR) tests for respiratory syncytial virus and COVID-19 were negative. An electrocardiogram (ECG) showed a paced rhythm.

**Figure 1:**
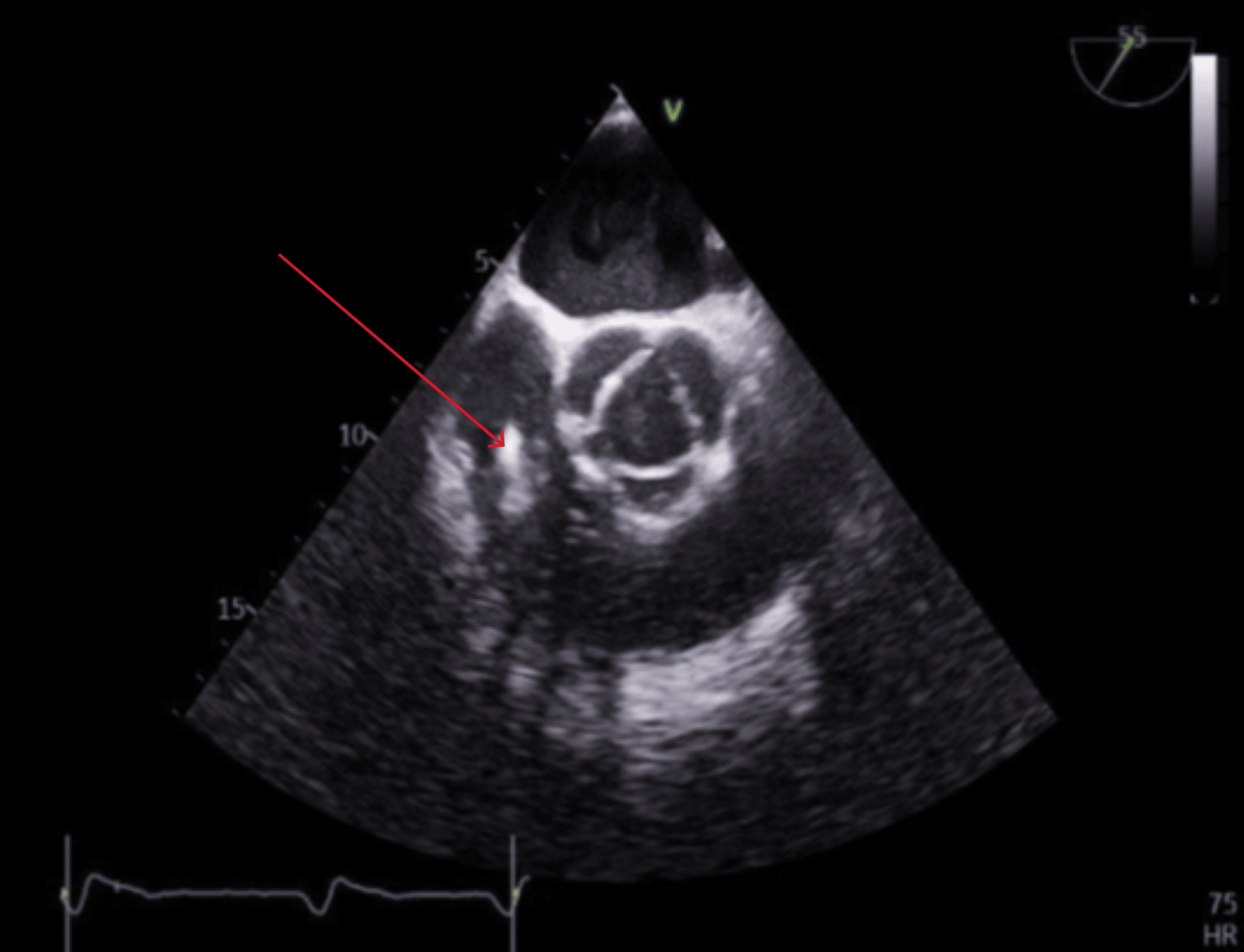
Transoesophageal echocardiogram showing echogenic shadowing of at least 2.5 by 1.3 cm in diameter on the right ventricular pacing lead consistent with vegetation.

Infectious serology tests for malaria, tuberculosis, Lyme disease, pneumococcal and legionella infections, HIV, hepatitis, *Coxiella*, and *Borrelia *returned negative. Additionally, chest X-rays and CT scans of the thorax, abdomen, and pelvis appeared unremarkable. While a transthoracic echocardiogram showed no significant findings, further evaluation via transoesophageal echocardiography (TOE) revealed an echogenic shadowing near the pacing lead in the right ventricle, measuring at least 2.5 by 1.3 cm in diameter. This finding indicated vegetation on the ventricular pacing lead, suggesting a likely diagnosis of endocarditis of the ventricular pacing lead (Figure 1).

Therapeutic intervention

The patient initially received intravenous antibiotics, but we discontinued the treatment, and the subsequent blood culture was positive. A TOE confirmed the presence of vegetation in the pacemaker lead. Subsequently, a triple antibiotic therapy regimen, consisting of vancomycin and gentamicin, was initiated. Post sensitivity testing, vancomycin was discontinued, and the patient was prescribed flucloxacillin and rifampicin while continuing with gentamicin. The infected pacemaker was surgically removed, following which the patient underwent a four-week course of vancomycin treatment, and a new pacemaker was inserted.

Follow-up and outcomes

The subsequent blood culture was negative following the completion of four weeks of antibiotics. A new pacemaker was inserted, and the patient has remained asymptomatic since the removal of the old pacemaker lead. The patient was discharged safely from the hospital and is due to be followed in the outpatient pacing clinic.

## Discussion

Infections involving ICDs and pacemaker leads pose serious health risks, especially when associated with endocardial infection, with mortality rates up to 35% [4]. The discussion includes a case of pacemaker-lead infection caused by coagulase-negative staphylococci (CoNS), reviews CoNS microbiology, explores risk factors and prevention strategies for ICD infections, and outlines current diagnostic and management guidelines.

ICD contamination can occur during manufacturing, packaging, implantation, surgical site infection, spread from other body sites, or through skin erosion. Asymptomatic colonisation by normal skin bacteria may progress to infection [3]. The most common pathogens in ICD infections are *Staphylococcus aureus* and CoNS, occurring both within and beyond one year after implantation [5].

Infections caused by CoNS increasingly burden healthcare systems, primarily due to the widespread use of implanted medical devices [4]. Staphylococcus epidermidis, in particular, is associated with chronic infections, attributed to its ability to form small colony variants (SCVs) that persist intracellularly and demonstrate reduced antibiotic susceptibility [6]. Additionally, certain staphylococcal species can survive on the plastic sheaths of pacemaker leads without external nutrients by producing a biofilm-like, slimy matrix [7]. This biofilm plays a critical role in protecting the bacteria from host immune responses and antimicrobial agents, thereby facilitating persistent colonisation [7]. These factors contribute to the difficulty of managing lead infections with conservative therapy alone, often necessitating device extraction [8].

The incidence of ICD infection has shown a significant recent increase, attributed to a rise in device implantation [6, 7]. Risk factors for ICD infection encompass diabetes mellitus, end-stage renal disease, chronic obstructive pulmonary disease, corticosteroid use, history of prior device infection, renal insufficiency, heart failure, malignancy, pre-procedure fever, anticoagulant use, and skin disorders [6]. Mitigating factors for ICD infection include ensuring ICD insertion occurs in a suitably ventilated, equipped, and sanitised environment [7]; having generator changes executed by a skilled operator [8]; abstaining from temporary transvenous pacing prior to permanent ICD implantation [9]; and deferring elective ICD implantation in the presence of systemic infection indicators [4].

Diagnosing infective endocarditis in patients with ICEDs is particularly challenging due to the lower accuracy of echocardiography and reduced sensitivity of blood cultures, increasing the risk of missed or delayed diagnosis [4].

Although the modified Duke criteria’s effectiveness for diagnosing ICED-related infective endocarditis or lead infection is unproven [5, 6], they remain a key tool for clinical assessment. Negative blood cultures are more common in ICED infections than in native valve endocarditis, but do not rule out infection. UK data highlight the importance of echocardiography in all patients with an ICED and Staphylococcus aureus bacteremia [7]. This case emphasises the critical role of TOE in suspected pacemaker-lead infections, given the low sensitivity of blood cultures and TTE [4]. Thus, TOE is recommended for ICED patients with unexplained fever and negative initial tests.

The case aligns with updated guidelines [10] that combine the modified Duke and 2015 ESC criteria for diagnosing ICED infective endocarditis [7, 11]. According to these guidelines, a definitive diagnosis requires either two major criteria or one major plus three minor criteria. Major criteria include microbiological or imaging evidence of ICED infection or infective endocarditis, while minor criteria encompass factors such as predisposing heart conditions, fever over 38°C, vascular phenomena, positive blood cultures not meeting major criteria, and serological or culture evidence from device pockets or leads [7, 11].

The most notable changes to the modified Duke criteria are, firstly, organisms such as CoNS, *Corynebacterium striatum*, *Corynebacterium jeikeium*, *Serratia marcescens*, *Pseudomonas aeruginosa*, *Cutibacterium acnes*, non-tuberculous mycobacteria, and *Candida* spp. are considered major clinical criteria in the diagnosis of infective endocarditis in the presence of ICEDs. Secondly, a major imaging criterion related to 18F-fluorodeoxyglucose (FDG) PET/CT has been included, and findings concerning native valve, cardiac device, or prosthetic valve more than three months post cardiac surgery are considered equivalent to echocardiography. Lastly, ICED implantation is among the minor criteria for predisposing factors [12].

The primary treatments for ICED infections are device removal and antimicrobial therapy. Device extraction is crucial and significantly improves outcomes, with studies showing that relying on antibiotics alone leads to a nearly sevenfold increase in 30-day mortality [13]. Early removal, ideally within 72 hours of hospital admission, is associated with reduced in-hospital mortality and shorter stays [14,15]. However, performing the procedure in the presence of systemic infection may be linked to higher mortality, warranting careful clinical judgement [16].

For blood culture-positive ICED infective endocarditis caused by methicillin-susceptible *Staphylococcus aureus *with lead or valve vegetations, the 2015 European Society of Cardiology (ESC) guidelines [11] recommend a minimum six-week course of flucloxacillin or oxacillin, combined with rifampin and once-daily gentamicin to minimise renal toxicity. The decision to reimplant a pacemaker should be carefully evaluated, with reimplantation delayed until infection has resolved and blood cultures are negative, either for at least 72 hours post extraction if no vegetations are present, or for two weeks if vegetations are identified [17]. This case supports such an approach, as reimplantation occurred only after symptom resolution and negative cultures.

## Conclusions

The case highlights that *Staphylococcus epidermidis*, a CoNS, can cause delayed pacemaker lead infections with a subtle, chronic course of infective endocarditis, making diagnosis and treatment difficult. Clinicians should maintain a high index of suspicion in patients with prolonged fever and perform TOE when TTE is inconclusive. Early and accurate diagnosis is critical to guide timely management, including the consideration of complete device removal to prevent further complications. Moreover, this case underscores the importance of multidisciplinary collaboration between cardiology, infectious disease, and cardiac surgery teams to optimise patient outcomes in complex device-related infections.
